# Cross-fusion of digital twins and artificial intelligence in diabetes: from mechanistic elucidation to full-cycle precision management

**DOI:** 10.3389/fendo.2026.1919404

**Published:** 2026-09-04

**Authors:** Yue Sun, Ruodi Yang, Qirui Xin, Xinxin Luo, Ying Zhou, Zishan Fu, Jiaxin Li, Zhichao Gu, Juntong Liu, Qingfeng Wang, Yufeng Yang

**Affiliations:** 1 Department of First Clinical College, Liaoning University of Traditional Chinese Medicine, Shenyang, China; 2 College of Acupuncture and Moxibustion, Liaoning University of Traditional Chinese Medicine, Shenyang, China; 3 Department of Teaching and Experiment Center, Liaoning University of Traditional Chinese Medicine, Shenyang, China; 4 Department of Basic Medical Sciences, Liaoning University of Traditional Chinese Medicine, Shenyang, China; 5 Department of Second Clinical College, Liaoning University of Traditional Chinese Medicine, Shenyang, China; 6 Department of Academic Affairs Office, Liaoning University of Traditional Chinese Medicine, Shenyang, China

**Keywords:** artificial intelligence, diabetes mellitus, digital twin, hybrid modeling, personalized management, precision medicine

## Abstract

The integration of digital twins (DT) and artificial intelligence (AI) is driving a paradigm shift in diabetes mellitus (DM) care from traditional population-based, symptomatic intervention to a lifecycle-oriented precision management approach. This review systematically elaborates on the cross-disciplinary integration mechanisms of the two technologies. By integrating multimodal data and constructing physiologically constrained hybrid models, it achieves multi-scale mechanistic analyses ranging from single-cell β-cell protection to multi-organ complications at the basic research level. On the clinical application front, it not only significantly enhances the early screening sensitivity for diabetic retinopathy and the accuracy of blood glucose prediction, but also optimizes insulin dosing, personalized nutritional plans, and exercise decision support through virtual trials. In the field of drug development, virtual clinical trials accelerate target discovery and drug repurposing. Although substantial technical, regulatory, and ethical challenges remain unresolved, ongoing progress in hybrid modeling, federated learning, explainable AI, and evolving regulatory frameworks provides a plausible pathway toward individualized prediction and proactive management, provided that claims of clinical readiness are matched by rigorous prospective validation.

## 1 Introduction

Diabetes mellitus (DM) is a metabolic disorder characterized by chronic hyperglycemia and is mainly classified into type 1 diabetes mellitus (T1DM) and type 2 diabetes mellitus (T2DM) (1, 2). According to the latest data released by the International Diabetes Federation (IDF) in 2025, there are 589 million adults aged 20–79 living with diabetes worldwide, corresponding to a prevalence rate of 11.11% — equivalent to 1 in every 9 adults having diabetes. By 2050, the number of people with diabetes is projected to increase to 853 million (3, 4). Together, these data indicate that diabetes has become a major global health challenge. Its associated complications, including cardiovascular disease, kidney disease, retinopathy, and neuropathy, also pose serious threats to patients’ quality of life and overall health (2, 5). However, current basic research on this disease largely relies on establishing *in vitro* cell models and *in vivo* animal models. *In vitro* cell models mainly include MIN6 pancreatic β-cells, HepG2 hepatocytes, and others, while the *in vivo* animal models commonly used include db/db mice, ob/ob mice, Zucker diabetic fatty (ZDF) rats, and non-obese diabetic (NOD) mice. Such models suffer from inherent problems such as species physiological differences, making it difficult to faithfully recapitulate the complex pathophysiological processes of human diabetes (6, 7). Furthermore, according to a report from the U.S. Food and Drug Administration (FDA), for some common diseases, medications fail to achieve the expected therapeutic effects in 38%–75% of patients (8). This highlights the significant gap between the population-based medical framework and individual complexity, making it difficult to truly translate research findings into clinical practice.

Digital Twin (DT) technology was originally created in the engineering field and was later gradually applied to other industries. A digital twin integrates information such as physical models, multi-source sensor data, and the full-lifecycle operational history of an object to construct a digital model in virtual space. This digital model is highly consistent with the real object in geometry, physics, behavior, and governing rules, is updated in real time, and interacts with and co-evolves with the physical entity (9).

In recent years, digital twin technology has been gradually applied to the medical field (10). The integration of digital twins and artificial intelligence promotes more precise and more personalized management of metabolic diseases. Medical digital twin technology faithfully reproduces the patient’s physiological state by constructing individualized models. Combining AI’s high-precision prediction and continuous adjustment abilities, DT-AI technology breaks through the limitations of traditional models that are static in time and homogeneous in population, constructing models that are calculable, predictable and intervenable for patients. Virtual clinical trials conducted via medical digital twins are utilized in various scenarios such as surgical rehearsal and new drug development (11). This advanced technology brings value to advancing the field of precision diabetology (12).

However, at the current stage, the potential of this technology in diabetes research has not yet been fully realized, because most current research and diagnostic/treatment systems are based on isolated studies rather than integrated ones. For example, traditional approaches generally focus on symptomatic treatment, namely controlling the superficial symptom of hyperglycemia, while failing to deeply elucidate the roles of pathological mechanisms such as chronic low-grade inflammation, gut microbiota, and endocrine dysfunction of adipose tissue in the disease progression (13). Clinically, different complications are managed by different specialists. Although this division of labor is clear, it overlooks the interconnections among complications. Such a phenomenon can easily lead to one-sided diagnosis and treatment, and prevents physicians from gaining a comprehensive grasp of the patient’s condition (14). Therefore, we urgently need to break through the existing problems through technological innovation. This review will focus on the latest research progress, technical architecture, diagnosis and treatment, and application scenarios of digital twins and artificial intelligence in the field of diabetes, as well as discuss challenges such as ethics and algorithmic interpretability. Ultimately, we believe that this promising new paradigm will move from theory to clinical practice.

**Figure 1:**
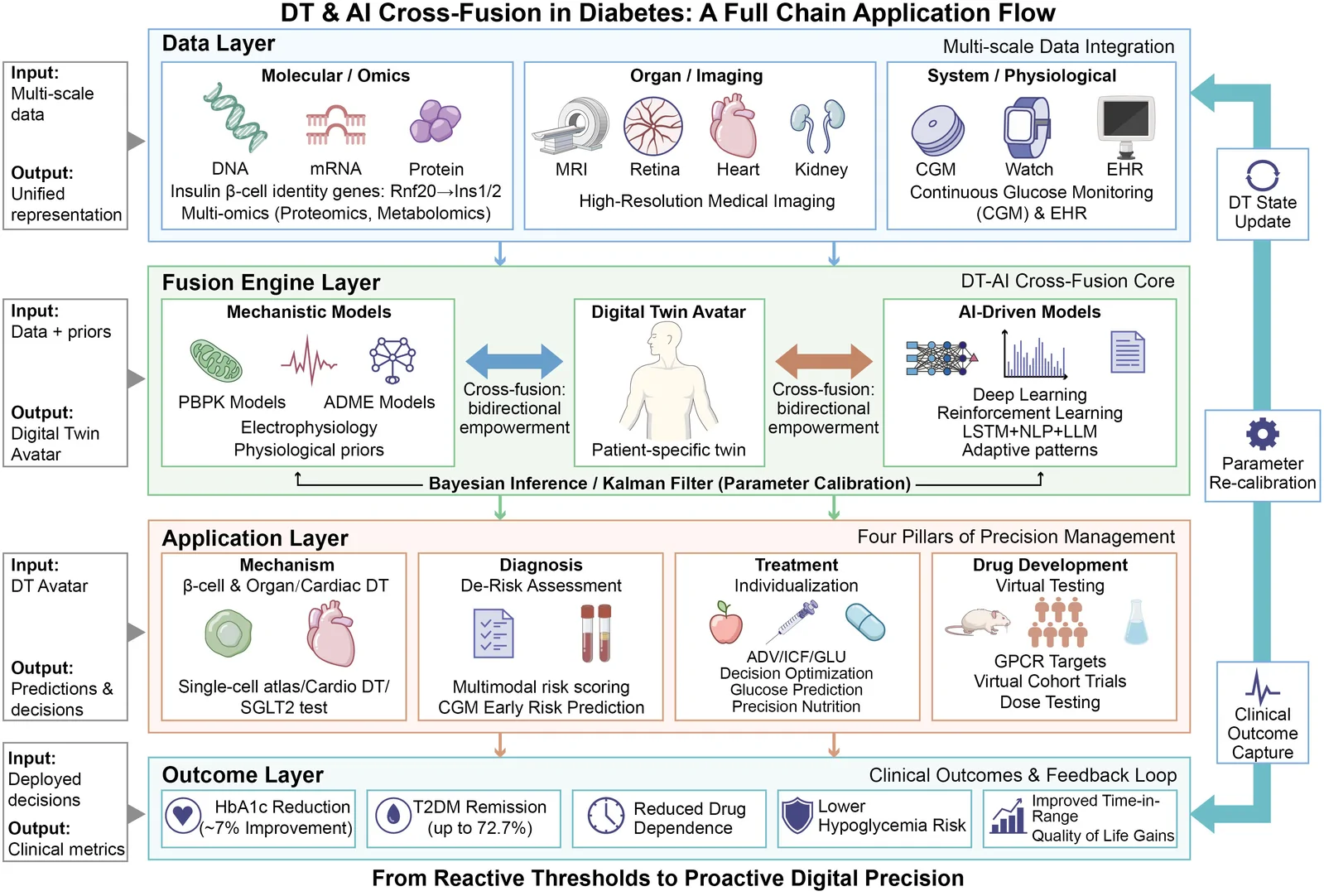
Full-chain application workflow of DT-AI cross-fusion in diabetes research. The figure summarizes how digital twin and artificial intelligence technologies support diabetes research across mechanistic modeling, precision screening, individualized treatment optimization, drug development, and clinical translation. It emphasizes multi-omics integration, real-time physiological monitoring, hybrid modeling, risk stratification, automated insulin delivery, insulin adjustment, glucose prediction, and improved HbA1c control.

Prior reviews on DT or AI in diabetes have largely treated the two technologies in parallel — Katsoulakis et al. (15) surveyed digital twins across health domains without a diabetes-specific integration lens; Kovatchev et al. (16) emphasized closed-loop control in T1DM through human–machine co-adaptation; and Chu et al. (17) focused on the medical digital twin framework and complication-risk prediction. What remains missing is an integrated account of how DT and AI reciprocally empower each other — the “cross-fusion” concept we advance here — and how this bidirectional synergy operates across the full diabetes lifecycle, from single-cell β-cell mechanism through complication prediction, precision diagnosis, individualized treatment, and drug development. This review therefore addresses three specific gaps: (i) it provides the first mechanistic decomposition of DT→AI and AI→DT empowerment pathways (Section 2.2); (ii) it maps DT-AI applications onto a full-cycle framework rather than treating them as isolated use-cases (Section 3 and **Figure 1**); and (iii) it provides a head-to-head critical comparison of representative DT studies in diabetes on dataset, technique, validation, and reported limitations (**Table 1**).

**Table 1:** Head-to-head comparison of representative digital-twin studies in diabetes.

Study	Population/dataset	AI/modeling technique	Validation strategy	Reported limitations	References
Visentin et al./UVA-Padova S2017	300 virtual patients across age groups; mixed-meal calibration	Multi-compartment ODE plus sensor-error model	V1 in silico validation; FDA-recognized context for artificial pancreas testing	Rigid exercise modeling; limited fat/protein meal handling	(18–20)
Young et al./exDSS	OHSU T1DM virtual patient population	Physiological ODE simulation plus rule-based exercise decision support	V1 in silico validation using standardized 30-minute exercise bouts	No real-world human validation; narrow exercise scenarios	(21)
Thamotharan et al./HDT	15 elderly T2DM patients; 14-day IoMT data	LSTM + Matrix Profile + LIME/XGBoost + LB-MPC	V3 small-cohort real-world observation	Small sample; short follow-up; LSTM opacity; no randomized closed-loop trial	(22)
Nemitz et al./dapagliflozin DT	Pooled data from 28 clinical PK/PD studies	PBPK plus renal glucose-regulation ODE	Cross-study PK/PD validation	Static covariate handling; limited to one drug class	(23)
Shamanna et al./Twin Precision Nutrition	Real-world T2DM cohorts with CGM and dietary data	Multimodal machine learning on CGM, lifestyle, and dietary logs	Retrospective real-world observation	Non-randomized; possible selection bias; limited causal inference	(24–26)

### 1.1 Literature search strategy

This work is presented as a narrative (non-systematic) review with semi-scoping features. Between January 2024 and July 2026, we searched PubMed, Web of Science, Scopus, IEEE Xplore, and Google Scholar. The core Boolean search string was: (“digital twin” OR “in silico patient” OR “virtual patient”) AND (“diabetes” OR “type 1 diabetes” OR “type 2 diabetes” OR “gestational diabetes” OR “prediabetes”) AND (“artificial intelligence” OR “machine learning” OR “deep learning” OR “hybrid model” OR “physics-informed neural network”). The time window covered publications from January 2015 to July 2026, with priority given to studies published after 2020. Inclusion criteria were: (i) peer-reviewed English-language original research, systematic reviews, or authoritative regulatory/consortium reports; (ii) explicit engagement with either DT or AI methodology relevant to diabetes; (iii) sufficient methodological detail to permit critical appraisal. Exclusion criteria were: (i) conference abstracts without full text; (ii) non-English publications; (iii) studies addressing chronic diseases other than diabetes without transferable methodology. From an initial 1,246 records, 187 were retained after title/abstract screening and 106 after full-text assessment; the final citation list further includes seminal landmark references identified through backward citation tracking (the screening numbers are summarized narratively below). We acknowledge the intrinsic limitations of a narrative approach — including potential selection bias, over-representation of high-impact positive findings, and possible underrepresentation of negative or null results — and have therefore made a deliberate effort to include studies reporting limitations and unresolved challenges (27).

## 2 Fundamental theories and technical basis

### 2.1 Digital twin technology

In practical terms, DT creates a computational replica of a physical object, with the two synchronized in structure, state, and behavior (15). In the field of diabetes research, a DT is a dynamic virtual replica created for an individual patient by integrating computational modeling and real-time data. This dynamic virtual replica has the characteristics of being updated synchronously with the disease progression and being highly tailored to the individual’s features (11). In diabetes research, this virtual representation can be updated in real time and used to support simulation, machine learning, reasoning, and clinical decision-making (11).

#### 2.1.1 Key technical characteristics of digital twins in medical research

##### 2.1.1.1 Personalization and dynamicity

DT is a digital avatar tailored to a specific patient. According to Katsoulakis et al., a medical digital twin can be viewed as a personalized virtual mirror. It integrates diverse data encompassing clinical, genetic, and molecular profiles, as well as environmental and social determinants, into a unified model, thereby enabling multidimensional analysis. This approach facilitates dynamic, *in silico* predictions of how different treatment regimens will affect the human body, coupled with continuous monitoring and forecasting of an individual’s health trajectory. Highly intuitive, it ultimately serves as a powerful tool for disease prevention and treatment (28).

##### 2.1.1.2 Multimodal data fusion

The first step in creating a medical digital twin is to integrate various types of data (29). Take diabetic retinopathy (DR) as an example. It is a common and relatively unique microvascular complication of type 2 diabetes mellitus (T2DM) (30). To build a digital twin model for DR, we need to collect imaging data such as color fundus photography, OCT angiography, and FFA, along with blood glucose dynamic information, thereby forming a complex model that integrates multimodal data. The digital twin modeling approach that integrates multimodal data is expected to significantly improve the early warning sensitivity for DR and also better predict disease dynamics (31).

##### 2.1.1.3 Prediction and simulation capabilities

Digital twins enable virtual experiments. They can clearly display a patient’s disease indicators, simulate how the condition evolves under different treatment regimens. Furthermore, it can predict whether unimplemented interventions might trigger adverse events (29). In the context of diabetes research, continuous streams of real-time physiological data from wearables, combined with periodic biomarker measurements, feed into the digital twin model. This facilitates dynamic health evaluation and, crucially, captures early signals of perturbed glucose homeostasis before overt symptoms emerge. This predictive capacity improves early diagnostic accuracy and liberates clinical practice from purely reactive responses, marking a major advance in active disease prevention (11).

##### 2.1.1.4 Closed-loop interaction

Digital twins leverage real-world clinical data for the continuous calibration of model parameters. The optimized predictive outputs are subsequently relayed back to clinicians and patients, whose post-intervention data is funneled back into the model, thereby establishing an iterative clinical-model-clinical feedback loop. In the field of diabetes, Kovatchev et al. implemented this concept in T1DM through a human–machine co-adaptation strategy. Using digital twin technology, they facilitated mutual adaptation between the automated insulin delivery (AID) system and the patient. This closed-loop mechanism effectively rectifies the inherent flaw of traditional AID systems — characterized by the algorithm’s unawareness of patient physiology and the patient’s lack of insight into algorithmic logic — ultimately leading to more efficient glycemic management (16).

#### 2.1.2 Commonly used digital twin modeling methods in diabetes research

In the field of diabetes research, the modeling methods of digital twin technology are developing rapidly, with the main trend shifting from traditional pure physical models to hybrid modeling deeply integrated with artificial intelligence (32). At the current stage, mainstream modeling methods can be categorized into three types: mechanistic models, data-driven models, and hybrid models. A detailed comparison of the above three modeling methods in terms of theoretical framework, mathematical form, key advantages, and limitations is shown in **Table 2**.

**Table 2:** Comparison of commonly used digital twin modeling methods in diabetes research.

Method category	Theoretical foundation	Mathematical form	Key advantages	Limitations	Typical applications	References
Mechanistic models	Physiological principles; reductionist modeling	ODE/PDE systems; compartmental models; PBPK/PK-PD models	High interpretability; strong biological plausibility; safer extrapolation; regulatory familiarity	Difficult individual parameter identification; limited ability to absorb noisy high-dimensional patient data	Closed-loop algorithm validation; drug-mechanism simulation; disease-progression modeling	(18–20, 33)
Data-driven models	Statistical learning and function approximation	Neural networks; LSTM/Transformer models; random forest/XGBoost	Strong nonlinear mapping; suitable for CGM, imaging, and wearable data streams; adaptable to individual heterogeneity	Limited interpretability; weak out-of-distribution generalization; high data demand	Glucose prediction; diabetic-retinopathy screening; risk scoring; insulin-dose decision support	(30, 31, 34, 35)
Hybrid models	Physiology-informed learning and inductive bias	PINNs; Bayesian hierarchical models; mechanistic-AI fusion	Balances physiological plausibility and flexibility; reduced data hunger; improved interpretability compared with black-box AI	High engineering complexity; computational cost; lack of diabetes-specific benchmarks	Personalized DTs; T2DM trajectory prediction; multi-scale mechanism-to-clinic modeling	(32, 36–38)

**Figure 2:**
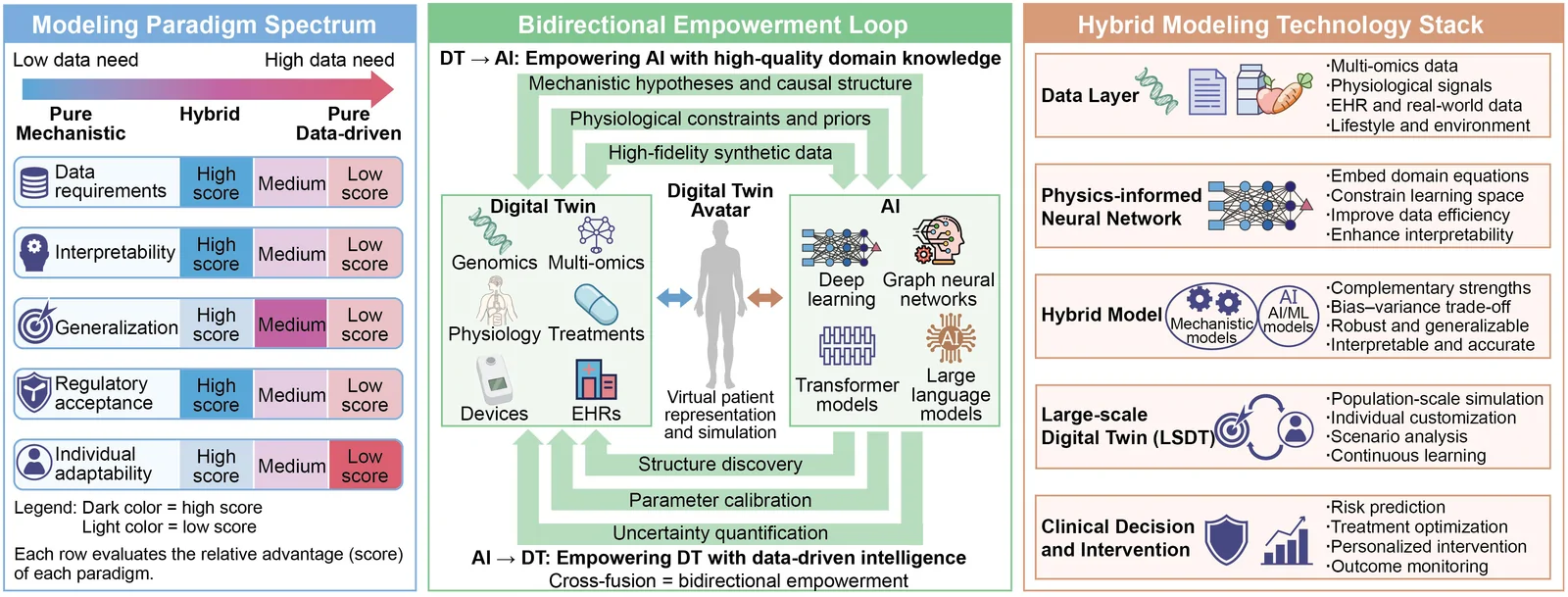
Bidirectional DT-AI empowerment and hybrid modeling architecture. The figure illustrates the reciprocal relationship between digital twins and artificial intelligence. Digital twins provide physiologically constrained virtual environments, synthetic data, and mechanistic priors for AI model development, whereas AI supports feature extraction, parameter calibration, prediction, and adaptive updating of digital twins. The figure also contrasts mechanistic, data-driven, and hybrid modeling paradigms.

A critical appraisal of these three paradigms reveals distinct trade-offs that have direct implications for diabetes DT design. Mechanistic models (e.g., the UVA/Padova simulator) offer regulatory-grade interpretability and strong extrapolation safety, but suffer from parameter-identifiability challenges when personalized to individual patients — a particularly acute problem in T2DM where inter-patient heterogeneity is high. Data-driven models excel at capturing individual heterogeneity from continuous glucose monitoring (CGM) and wearable data streams, but generalize poorly across sites and populations, and their opacity limits clinical adoption. Hybrid models — particularly physics-informed neural networks (PINNs) — currently represent the most promising direction for diabetes DTs because they preserve physiological plausibility while retaining machine learning’s flexibility for individualization. However, PINNs demand significant computational and engineering resources, and standardized benchmarks for diabetes-specific hybrid models do not yet exist. Comparative studies also indicate that no single paradigm dominates across all diabetes subtypes: mechanistic approaches remain the reference standard for T1DM closed-loop control, whereas hybrid approaches are gaining ground in T2DM long-term trajectory prediction and in gestational diabetes mellitus (GDM) risk stratification (36). This gap in unified benchmarking is a key barrier to broader adoption and is a priority in the roadmap outlined in Section 5.3. The corresponding modeling spectrum and bidirectional DT-AI empowerment architecture are summarized in **Figure 2**.

### 2.2 Cross-fusion mechanism of DT and AI

In the healthcare field, artificial intelligence is represented by machine learning, deep learning, and large language models. Artificial intelligence can automatically identify massive, high-dimensional, and multi-type data while continuously optimizing its learning, thereby achieving a full set of intelligent capabilities including understanding, reasoning, prediction, and decision optimization. It can then facilitate a paradigm shift in key areas such as computer-aided disease diagnosis, risk prediction, and personalized treatment (34). As the population of diabetic patients continues to expand, artificial intelligence technology is gradually being deeply applied to various scenarios of chronic disease management (34, 35). The collaboration between DT and AI in the field of diabetes has formed a deeply integrated relationship characterized by bidirectional empowerment and co-evolutionary synergy.

#### 2.2.1 Digital twins empowering artificial intelligence

The essence of digital twins empowering artificial intelligence is to construct a virtual metabolic environment constrained by physiological laws (39). Specifically, the role played by digital twins in this context is reflected in three aspects: virtual trials for data generation and model rehearsal; physiological constraints ensuring that the learning process does not deviate from human physiological laws; and knowledge integration combining scientific principles with real-world data. First, based on mechanistic physiological models such as the UVA/Padova simulator, digital twins can simulate clinically rare and critical situations, such as severe hypoglycemia and acute complications during pregnancy. They can also generate a statistically representative virtual patient population, providing artificial intelligence with abundant and reliable synthetic data, thereby overcoming the bottleneck of AI’s reliance on real-world data (33). Secondly, many models that simulate blood glucose dynamics in diabetic patients overlook human physiological mechanisms, resulting in model performance at the individual level being far inferior to population average metrics. One study proposed a physiologically constrained neural network digital twin approach that adheres to biophysical laws to simulate glucose fluctuations in patients with T1DM, thereby ensuring both model interpretability and physiological fidelity (40). Furthermore, conventional AI approaches often struggle to integrate scattered and fragmented knowledge sources. In contrast, the Large Language Model–Augmented Semantic Digital Twin (LSDT) framework synergistically combines large language models (LLMs) with digital twins. The results demonstrate that this architecture enables the integration of implicit textual knowledge with explicit sensor data; moreover, operating at a semantic level allows the digital twin to transcend mere rule adherence and facilitate intelligent decision-making (41).

#### 2.2.2 Artificial intelligence empowering digital twins

Traditional digital twin models have the shortcomings of structural rigidity and difficulty in individual adaptation. When a patient presents with special circumstances not pre-defined in the model, such as a specific genotype or rare complications, attempting personalized calibration of the model may lead to problems such as insufficient parameter identifiability or difficulties in fitting (42). Machine learning algorithms, represented by deep neural networks, can extract complex features from large amounts of real-world data and identify hidden factors that are not explicitly specified in the mechanistic models of digital twins but nevertheless exist in reality, such as the effects of mental stress, sleep, and the menstrual cycle on pancreatic islet function (33, 43, 44), and, through residual learning or parameter adaptation methods, enable the model to achieve continuous personalized calibration (43). Furthermore, clinical trust is crucial. According to a survey, although digital twin technology has gained widespread attention among healthcare professionals, only 30% to 40% are able to confidently interpret and act upon it without systematic training (29, 45). Here, attention mechanisms, counterfactual explanations, and Local Interpretable Model-agnostic Explanations (LIME) serve as pivotal explainable AI (XAI) techniques. These methods collectively establish the technical foundation for transforming artificial intelligence into a clinically trustworthy tool. Although the practical application of XAI technology is still limited by multiple real-world factors, significant progress will be achieved in the future as new breakthroughs are made in theory, technology advances, and applications are gradually implemented (11).

#### 2.2.3 Collaborative evolution

The synergy between digital twins and artificial intelligence facilitates explainable, predictable, and dynamically evolving personalized management. Digital twins provide a safe, controllable virtual sandbox for the prospective evaluation of individualized treatment regimens and the prediction of their efficacy and risks. Using the artificial pancreas as an exemplar, this approach enhances glycemic monitoring and automates insulin delivery via algorithmic calculations, thereby empowering patients with T1DM to achieve superior glucose control (39). Meanwhile, artificial intelligence drives learning and inference within the virtual physiological environment established by the digital twin. Through extensive *in silico* trials, AI optimizes therapeutic regimens and translates these insights back into clinical practice (46). They engage in bidirectional empowerment, mutually reinforcing their respective strengths.

## 3 Application of DT-AI cross-fusion in diabetes research

### 3.1 Mechanism research of diabetes

Diabetes is a metabolic disorder characterized most typically by persistently elevated blood glucose levels, and its pathogenesis is essentially attributable to defective insulin secretion and/or impaired insulin function (47). Building on the multiscale integration principle established in Section 2.2, the DT-AI paradigm allows diabetes mechanism research to progress from isolated single-gene or single-pathway studies (48) to system-level analyses spanning genes, proteins, cells, tissues, and organs (49).

#### 3.1.1 Cellular level

Protecting pancreatic β-cells is of paramount importance in diabetes management, because they are the only cells that secrete insulin. Whether blood glucose can be controlled depends critically on the proper function of pancreatic β-cells (50). Most past studies have relied on static *in vitro* experiments or tissue sections (51), making it difficult to clearly understand how β-cells respond in real time within the physiological environment of the human body. To address this issue, building a digital twin of pancreatic β-cells is a promising strategy. By integrating multi-omics data with real-time metabolic monitoring information, and combining mechanistic models with machine learning algorithms, it is possible to simulate the dynamic changes in β-cell function with high accuracy. Elucidating the molecular basis of β-cell identity is critical to the development of high-fidelity digital twins. In a cell-line-based β-cell biology study relevant to T1DM and T2DM, Pierre et al. demonstrated that the Rnf20 protein, as an interacting partner of Islet-1 and an E3 ubiquitin ligase, ensures the normal expression of several key β-cell identity genes, including Ins1/2, MafA, and Pdx1, by maintaining an active chromatin state characterized by enrichment of H2Bub1. If Rnf20 is lost, β-cells undergo dedifferentiation, manifested by downregulation of identity genes and aberrant expression of the endocrine progenitor markers Ngn3 and Sox9. This finding is particularly critical, as it provides key quantitative parameters and regulatory targets for modeling the molecular mechanisms of β-cell dedifferentiation in digital twin models (52).

#### 3.1.2 Complication mechanisms

Diabetes is a complex disease not caused by a single factor, but by multiple contributing elements. Its pathogenesis involves multidimensional triggers such as genetics, immune dysregulation, microbial infection, toxins, oxidative stress, and psychological stress. These interfering factors disrupt normal metabolic processes, leading to abnormal glucose regulation and insulin resistance. Moreover, as the disease progresses, it can cause dysfunction and complications in multiple organ systems, including the heart, kidneys, retina, and feet (17). Chu et al. proposed a mixed-cohort framework for medical digital twins and regarded diabetic complication-risk prediction as one of the core application scenarios of digital twins. Therefore, in addition to constructing a systemic digital twin for diabetes, independent organ-specific digital twins for organs such as the heart and kidneys can also be built, with the aim of preventing and treating complications (17).

Constructing a cardiac digital twin to analyze diabetic heart disease represents a new perspective. Diabetic cardiac damage is a continuous pathological continuum, initiating with myocardial metabolic abnormalities and electrophysiological disorders, and eventually progressing to structural remodeling of the ventricles. With the aim of understanding this pathological process, researchers developed a patient-specific cardiac digital twin model for T2DM by integrating multiphysics data, including hemodynamics, electrical impulse conduction, and myocardial mechanics; this study used in silico validation. The advantage of this model is that it does not rely on a systemic metabolic model; it can independently simulate cardiac function and diabetes-induced cardiac remodeling, as well as test the direct cardioprotective mechanisms of glucose-lowering drugs such as SGLT2 inhibitors (53). This study is important because it addresses a mechanistic gap that is difficult for traditional clinical trials to uncover.

Taken together, the β-cell and complication-organ studies above illustrate two contrasting strategies for mechanistic DT construction: molecular-level modeling anchored in multi-omics data, versus organ-level modeling anchored in multiphysics data. Each has clear strengths — the former provides mechanistic granularity, the latter provides clinically translatable readouts — but neither has yet been prospectively validated in living patients, and cross-scale integration between them remains largely aspirational. This unresolved coupling between molecular and organ-level DTs is, in our view, the most important near-term research gap in diabetes DT mechanism research.

### 3.2 Precision diagnosis and risk prediction

#### 3.2.1 Limitations of traditional screening methods

Before discussing traditional diabetes screening methods, we need to clearly recognize a very serious problem: nearly half of the diabetic patients worldwide remain undiagnosed and only seek medical care when they develop severe symptoms or complications, thereby missing the optimal window for clinical treatment (4). Currently, the screening of diabetes and prediabetes mainly relies on several methods: fasting plasma glucose (FPG), oral glucose tolerance test (OGTT), glycated hemoglobin (HbA1c), and random blood glucose (47). Although these examination methods are well-established and widely used in clinical practice, their most prominent problem is that they still operate within the old framework of “threshold diagnosis” and represent a typical static diagnostic approach. Taking HbA1c as an example, its limitations manifest in three primary ways. First, there is the glycation gap — two individuals with identical glucose levels may exhibit markedly divergent HbA1c readings. Second, HbA1c is subject to significant inter-individual variability based on race, age, and sex. Third, it suffers from insufficient sensitivity; subtle initial elevations in blood glucose often go undetected by HbA1c assays. Collectively, these shortcomings contribute to missed diagnoses and misdiagnoses in clinical settings (54). Furthermore, different international organizations have markedly different diagnostic criteria for prediabetes, and no unified standard exists. These organizations include the American Diabetes Association (ADA), the International Diabetes Federation (IDF), Diabetes UK, and the Chinese Diabetes Society (CDS), among others (55). This leads to completely different diagnostic outcomes for the same individual under different diagnostic methods and guidelines, which is very unfavorable for clinical research on diabetes.

#### 3.2.2 Leveraging DT-AI for precision screening of diabetes

To effectively manage diabetes, it is essential to initiate standardized diagnosis, treatment, and risk prediction processes as early as possible (56). The combination of DT-AI can precisely address the limitations mentioned earlier. Digital twins serve as a data integration framework, bringing together scattered multidimensional information to achieve a comprehensive, dynamic virtual representation of each patient’s metabolism. Artificial intelligence acts as a recognition engine, extracting from it early disease signals that are difficult for humans to detect, thereby enabling precise diagnosis and risk prediction. Studies have shown that digital twin technology, particularly physiological modeling driven by machine learning, holds broad application prospects in the early diagnosis of diabetes and the realization of precision glucose intervention (37). In predicting gestational diabetes, a DT-AI model for GDM incorporating clinical parameters such as maternal characteristics, first-trimester biomarkers, and early pregnancy blood glucose measurements can identify pregnant women at high risk of gestational diabetes before 24 weeks, i.e., before the traditional screening window has even opened in a retrospective real-world validation study. This approach is more sensitive than traditional screening methods, and earlier detection means earlier intervention, which holds promise for preventing the onset of gestational diabetes (11). Second, medical imaging data are closely related to digital twins. High-resolution medical imaging techniques such as MRI, CT, and PET can provide accurate anatomical structures and physiological data without the need for surgical dissection or harming the human body, thereby providing a critical data foundation for constructing personalized digital twin models (57). One study has shown that humans can construct a “virtual retina twin” based on fundus images, where deep learning algorithms can autonomously identify DR staging from digital fundus images in T1DM and T2DM. In diabetic retinopathy screening, AI has been reported to achieve diagnostic performance comparable to that of ophthalmologists in specific validation settings. As a landmark achievement in this field, the IDx-DR system, in a pivotal trial involving 900 primary care patients, achieved 87.2% sensitivity, 90.7% specificity, and a 96.1% image acquisition rate, and became the first fully autonomous AI diagnostic system to receive FDA approval in a prospective clinical validation study (58). This regulatory milestone should nevertheless be interpreted with caution: FDA approval was granted for a narrowly defined screening indication in a primary-care setting, and does not imply that AI can operate independently across the broader spectrum of ophthalmological practice. In T2DM, Zhang et al. proposed a digital twin framework that integrates machine learning, multi-omics data, knowledge graphs, and mechanistic models. By identifying proteomic, metabolomic, and clinical features, it can effectively predict the evolution trend of multiple clinical variables of T2DM over a period of six months to one year in an in silico validation setting. The advantage of this framework is that it achieves continuous monitoring of patients and enables early prediction of dynamic changes in clinical variables (59). In summary, with the current state of technological development, we aggregate clinical data, imaging information, and multi-omics biomarkers within a digital twin framework, and then leverage artificial intelligence to extract early warning information from multidimensional data. This technological synergy is gradually transitioning diabetes care toward preventive precision medicine, opening up new pathways for improving the sensitivity and specificity of screening for diabetes and prediabetes.

### 3.3 Individualized treatment optimization

Tailoring treatment plans to each patient is the essence of personalized medicine, and the medical community has been exploring this for decades (29). DT-AI technologies are not only products of technological innovation but also promising key tools for revolutionizing personalized medicine. Digital twin technology does not merely focus on population statistics; instead, it integrates continuous glucose monitoring, wearable device data, electronic health records, and multi-omics data. It then employs data algorithms such as Bayesian inference and Kalman filtering to continuously infer and calibrate each individual’s true physiological parameters, bringing the virtual replica ever closer to the real human, and ultimately forming a dynamically updated, individualized digital life entity (34). The ADVICE4U clinical trial in T1DM illustrates the application of digital-twin concepts to insulin therapy and has undergone prospective clinical validation. It collects and analyzes real-time data including past blood glucose values, insulin doses, and carbohydrate intake, and then provides the most appropriate insulin dose recommendation for the individual at that moment (29, 60, 61). Furthermore, In T2DM, Paramesh Shamanna et al. constructed individualized digital twin models by integrating multimodal data such as continuous glucose monitoring and dietary logs, enabling dynamic prediction of the postprandial glycemic response after the patient consumes specific foods. In clinical practice, this supports a shift from generalized dietary prescriptions toward individualized nutritional recommendations. According to retrospective real-world data, the treatment effect reported is substantial: the HbA1c level dropped by nearly three percentage points, 94% of patients were able to stop relying on antihypertensive drugs, and 72.7% of patients achieved remission of T2DM within one year (24). A one-year retrospective real-world analysis of 1,853 individuals demonstrated meaningful glycemic control, reduced requirements for glucose-lowering agents, and notable improvements in overall metabolic health (25, 26). These improvements, while striking, should be interpreted with caution given the non-randomized design, potential selection bias in the enrolled cohorts, and the absence of an independent control arm; prospective randomized trials remain necessary before clinical readiness can be claimed. This case nevertheless suggests that when a nutritional plan is precisely matched to an individual’s unique metabolic characteristics, the effectiveness of diabetes management can be meaningfully improved.

A cross-study comparison of the individualized-treatment literature exposes a clear methodological gradient. The ADVICE4U trial illustrates a controlled prospective design in T1DM but at limited scale; the Shamanna et al. programme in T2DM offers real-world scale but lacks randomization; and most other reports remain single-arm observational studies. Two research gaps deserve emphasis: (i) the absence of head-to-head trials comparing DT-AI-guided regimens with best-practice standard care, and (ii) the underreporting of failure cases and negative outcomes, which may lead to publication bias and inflated effect estimates.

### 3.4 Diabetes drug development

To date, in addition to insulin, the FDA has approved 59 glucose-lowering drugs for clinical use: 36 for use as monotherapy and the other 23 for combination therapy (62). Established pharmacotherapeutic options for diabetes encompass sulfonylureas (SU), thiazolidinediones (TZD), biguanides, α-glucosidase inhibitors, dipeptidyl peptidase-4 (DPP-4) inhibitors, sodium-glucose cotransporter-2 (SGLT2) inhibitors, glucagon-like peptide-1 receptor (GLP-1R) agonists, and meglitinides (62, 63). The more drug options available, the more difficult the decision-making becomes. Faced with dozens of glucose-lowering drugs and numerous possible combinations, how to find the optimal regimen for each patient is a key challenge that precision medicine urgently needs to address. Moreover, traditional drug development for diabetes requires substantial time and cost, yet the returns often fall short of expectations. Taking T2DM as an example, despite massive resources invested over decades, the failure rate of new drug candidates in clinical trials remains high, and the number of approved disease-modifying therapies is still very limited (64). The judicious integration of DT-AI holds considerable promise for addressing these limitations.

#### 3.4.1 Drug target discovery

G protein-coupled receptors (GPCRs) are widely distributed in metabolic organs such as the pancreas, liver, muscle, adipose tissue, and intestine, and are directly involved in the regulation of blood glucose. Therefore, they have become key targets for diabetes drug development. Among all FDA-approved clinical drugs, more than 30% are related to GPCRs, with semaglutide and sitagliptin being representative examples (65). Boeringer et al. believe that the convergence of digital twins, quantitative systems pharmacology (QSP), and artificial intelligence will bring about a major innovation in GPCR-targeted therapy (66).

#### 3.4.2 Drug repurposing

Another function of digital twins is to quickly assess whether existing marketed drugs have new value in the treatment of diabetes. The effects of traditional drugs require extensive experimental screening, which is complex and time-consuming. With digital twin technology, by mapping drug mechanisms onto disease molecular networks, existing drugs with multi-pharmacological effects can be identified. This approach not only avoids the need to develop new drugs from scratch, but also benefits from the higher safety profile of established drugs. This strategy has been validated in the fields of oncology and neurodegenerative diseases (63), and its application in diabetes could also prove useful, for example in screening cardiovascular protective drugs that also have glucose-lowering effects.

#### 3.4.3 Clinical trial optimization

Building on the recruitment, cost, and cycle-length constraints noted above (67, 68) DT-AI can generate a statistically representative virtual patient population from real-world data. Researchers can conduct extensive trials within this virtual patient population to obtain drug responses and risks across different patient groups. This approach can reduce the uncertainty of human studies and decrease the scale and cost of physical trials (45). For some rare diseases, the number of patients is inherently small, so the pool of eligible trial participants is naturally limited, making it impossible to enroll a sufficient sample size to demonstrate whether a drug is effective (69). By using real patient databases or historical clinical trial data, digital twin technology can create virtual cohorts matched to the target population, thus effectively solving the problem of insufficient sample size (70).

#### 3.4.4 Individualized dosing determination

Most clinicians adjust the dosage of glucose-lowering drugs for patients based on population-level medication protocols or their own clinical experience, while differences among patients in terms of insulin sensitivity, pancreatic β-cell function, pharmacokinetics, and lifestyle factors are not given sufficient attention (71). Using a mixed T2DM cohort that included a chronic kidney disease subgroup, Nemitz et al. constructed and cross-validated a pharmacokinetic/pharmacodynamic digital twin for dapagliflozin. The model incorporated data from 28 clinical studies and accounted for inter-individual differences in factors such as renal and hepatic impairment and food intake, so that the patient’s medication dosage can be better tailored to their individual situation. The distinctive advantage of this dapagliflozin digital twin resides in its seamless integration of absorption, distribution, metabolism, and excretion (ADME) processes with the renal glucose regulatory mechanisms. Notably, the model demonstrates that renal impairment significantly attenuates urinary glucose excretion, a finding that underscores the importance of personalized dosing strategies for patients with chronic kidney disease (CKD) (23).

Taken together, the four drug-development sub-directions above reveal an important asymmetry: while DT-AI is already producing measurable value in dose individualization (e.g., the dapagliflozin PK/PD twin) and in silico trial design (e.g., UVA/Padova-based virtual cohorts), its contribution to *de novo* target discovery in diabetes remains largely conceptual. This asymmetry has not been explicitly addressed in prior reviews. We argue that the near-term translational payoff lies in dose personalization and virtual-cohort augmentation, whereas target discovery and drug repurposing require substantial improvements in multi-omics annotation and causal inference before they can rival established wet-lab pipelines.

To translate the aforementioned theories into concrete clinical practice, this article constructs a full-chain application framework for the cross-fusion of digital twins and artificial intelligence in diabetes (as shown in **Figure 1**). The process begins with the integration of multi-scale data, which, by combining mechanistic models (e.g., PK/PD) with AI-driven models (e.g., deep learning), establishes a core digital twin capable of bridging molecular mechanisms and systemic physiology. This integrated system further supports full-cycle applications ranging from mechanistic elucidation, precision diagnosis, and individualized treatment to drug development, ultimately forming a closed-loop feedback system that shifts from reactive thresholds to proactive digital precision, thereby achieving more comprehensive full-cycle precision management.

## 4 Case analysis

To better illustrate how digital twins and artificial intelligence work together in diabetes research and how they transition to clinical practice, we selected three representative cases: the UVA/Padova T1DM simulator, a digital twin-based exercise decision support system (exDSS), and a human digital twin system (HDT) for elderly patients with type 2 diabetes mellitus (T2DM). Next, we will analyze each case from five dimensions: background, technical architecture, validation results, clinical value, and limitations.

**Figure 3:**
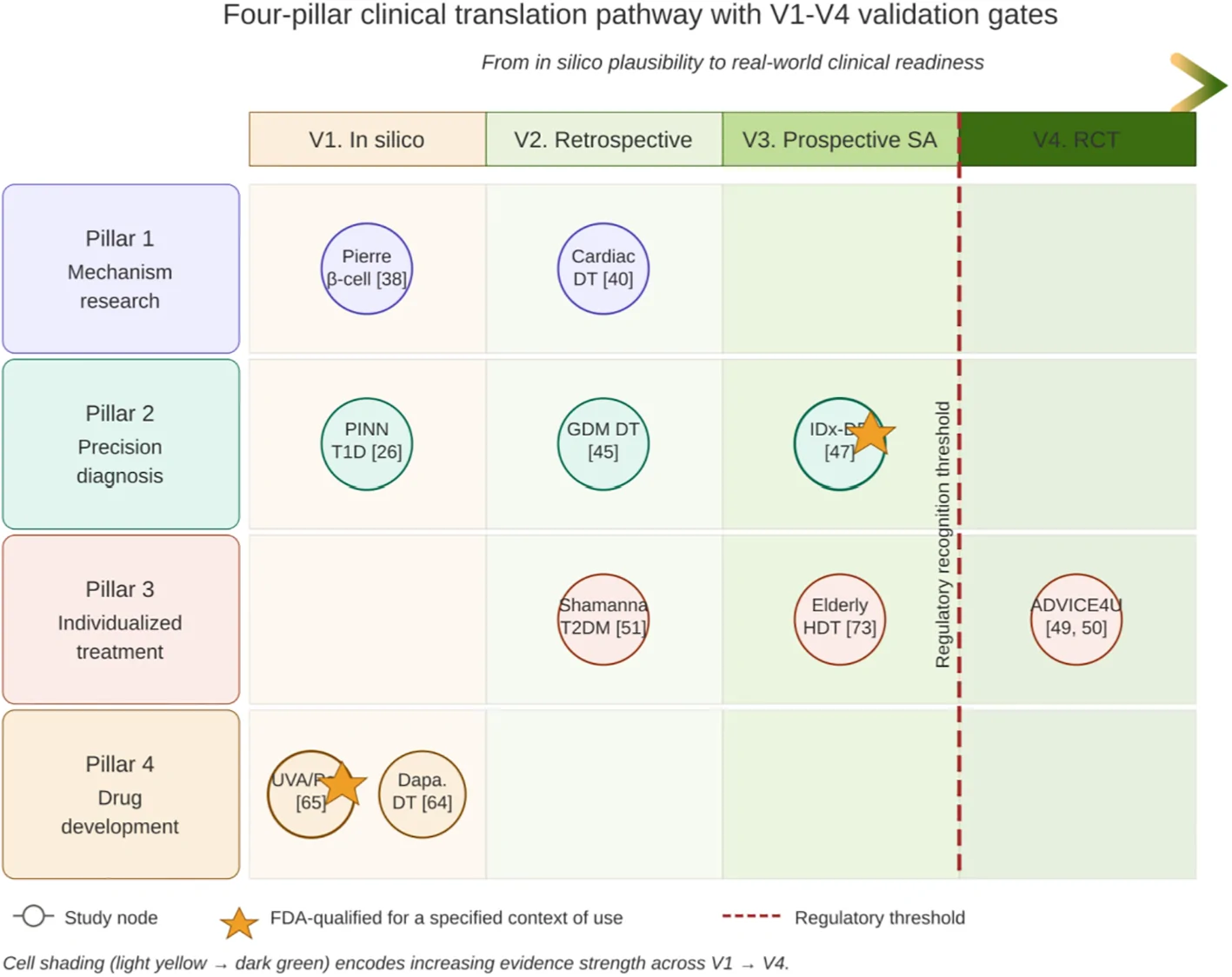
Four-pillar clinical translation pathway with V1-V4 validation gates. The figure summarizes a staged validation framework for representative diabetes digital-twin applications, moving from in silico validation to small-cohort real-world observation, prospective single-arm studies, and randomized controlled trials. Representative examples include the UVA/Padova simulator, dapagliflozin digital twin, PINN-based T1DM modeling, ADVICE4U, and the elderly T2DM human digital twin.

Before analyzing the three exemplar cases, we emphasize a crucial distinction that has sometimes been blurred in the literature: simulation-based (in silico) validation — where model outputs are compared against synthetic virtual patients or historical datasets — is fundamentally different from real-world clinical validation — where a system is prospectively tested in patients under closed-loop conditions. Only the former is available for exDSS; the HDT achieved a modest 14-day open-loop real-world observation; the UVA/Padova simulator, despite FDA recognition as a regulatory tool, has never been used as a therapeutic device on patients. We therefore refrain from claims of “clinical readiness” for any of these systems and instead frame their contributions in terms of translational potential. The same validation hierarchy is summarized in **Figure 3**.

### 4.1 UVA/Padova type 1 diabetes mellitus simulator: the evolution from single-meal to full-day coverage

Since receiving FDA recognition in 2008, the UVA/Padova T1DM simulator, an FDA-recognized regulatory tool validated only in silico, has become the recognized gold standard for artificial pancreas algorithm validation (18). However, earlier iterations of the simulator, specifically the 2008 and 2013 versions, failed to capture essential physiological rhythms such as the intraday variability in insulin sensitivity and the dawn phenomenon. Consequently, consuming the same meal at breakfast, lunch, or dinner would yield an identical glucose profile, irrespective of the time of day (18, 19). The S2017 version, introduced by Visentin et al., overcame this limitation. By incorporating intraday variability in insulin sensitivity, a dawn phenomenon model, multiple insulin pharmacokinetic profiles, and a sensor error model, the S2017 UVA/Padova T1DM simulator extended its scope from single-meal to full-day simulations, successfully reproducing circadian glucose dynamics. This version was calibrated using a three-tracer mixed-meal tolerance test. The simulator generated a cohort of 300 virtual patients spanning three age groups: children, adolescents, and adults. Notably, the simulated intraday glucose trajectories closely aligned with real-world continuous glucose monitoring (CGM) data. Technologically, the in silico testing platform circumvents the need for patient recruitment, ethical approval, and protracted time commitments, thereby eliminating patient risk and substantially reducing the time and financial investment required for preclinical evaluation. Clinically, by generating a virtual patient population that spans diverse age groups and metabolic phenotypes, the model facilitates the development of tailored algorithms for distinct subpopulations. From a regulatory science perspective, this marks the first instance of the U.S. Food and Drug Administration (FDA) authorizing the use of digital twins to replace certain animal studies. This milestone paves the way for the broader implementation of the in silico clinical trial paradigm across various disease domains. The current version still exhibits several limitations, notably the lack of granularity in modeling exercise effects, insufficient characterization of the impact of fat and protein on postprandial glucose excursions, and a failure to account for real-world variability in lifestyle factors such as diet, sleep, and mood. In the future, by integrating multi-omics data and machine learning technologies, it is expected to achieve an expansion from a single-day to a multi-day scale, thereby laying a more solid digital twin foundation for next-generation artificial pancreas systems, clinical decision support systems, and personalized precision therapy (20).

### 4.2 exDSS for T1DM: turning exercise from a “blood glucose risk” into a controllable variable

For patients with T1DM, long-term adherence to regular exercise is particularly important for improving their condition (72). However, the problem is that exercise affects blood glucose fluctuations. During aerobic exercise, the body’s tissues increase their demand for glucose, which can sometimes exceed the body’s own regulatory capacity, leading to a drop in blood glucose and a risk of hypoglycemia. Many patients are afraid to adhere to regular exercise precisely because they fear experiencing hypoglycemia (73, 74). Young et al. proposed a digital twin-based exercise decision support system (exDSS), validated only in silico, that provides personalized treatment recommendations before aerobic or resistance exercise. The system uses heart-rate, insulin-dose, and dietary data with an individualized model to simulate blood glucose dynamics during and after exercise, helping patients with diabetes exercise more safely and avoid hypoglycemia. This study used in silico validation. During aerobic exercise, without intervention, the time that blood glucose remained within the safe range was only 80.2%. However, with the exDSS tool, this proportion increased to 92.3%. Similarly, during resistance exercise, the time in the safe range rose from 72.3% to 87.3%. Regarding hypoglycemia risk, during aerobic exercise, the use of exDSS reduced hypoglycemia time from 15.1% to 5.1%; during resistance exercise, the time spent in hypoglycemia decreased from 18.2% to 6.6%. These in silico results suggest the favorable performance of exDSS. It is worth noting that, to the authors’ knowledge, exDSS is the first digital twin decision support system specifically designed for exercise scenarios in patients with T1DM. It may help generate safer and more effective exercise plans, reducing their tendency to avoid exercise due to fear of hypoglycemia. However, the current system still has limitations. Because the study was based only on in silico simulations, the effect estimates may be overly optimistic when extrapolated to real-world conditions. It was only validated for standardized 30-minute exercise sessions and cannot cover random real-life scenarios. Key directions for future development include improving the system’s practicality and generalizability: introducing reinforcement learning to optimize decision-making, integrating real-time data such as heart rate, mood, and environment; conducting comprehensive validation in more diverse and complex exercise scenarios; and performing short-term human studies to assess feasibility in real-world settings. In summary, this research demonstrates that digital twin technology may effectively support personalized glycemic management in standardized exercise scenarios. However, systematic validation will be required before clinical implementation (21).

### 4.3 Human digital twin: personalized management of elderly type 2 diabetes mellitus

Elderly patients with T2DM (E-T2D), due to their unique physiopathological characteristics such as decreased physical function, memory decline, polypharmacy, and the presence of multiple chronic comorbidities, significantly increase the difficulty of glycemic management (75). Thamotharan et al. developed a novel human digital twin system (HDT), evaluated in a small-cohort real-world observation, specifically designed for personalized glycemic management in elderly patients with T2DM. The HDT framework consists of four modules: a data module, a prediction module, a diagnosis module, and a management module. Multimodal data were acquired via an Internet of Medical Things (IoMT) architecture. A Long Short-Term Memory (LSTM) network performed multi-step glucose prediction from multivariate time-series inputs. Subsequently, a matrix profile algorithm integrated with explainable AI techniques (LIME and XGBoost) was employed to characterize glucose fluctuation patterns and perform risk attribution. Finally, a learning-based Model Predictive Controller (LB-MPC) computed the optimal insulin delivery dosage. The team recruited 15 eligible elderly patients with T2DM and collected data continuously for 14 days. They found that after using the HDT, the patients’ time in range (TIR) increased from 3%–75% to 86%–97%, insulin dosage decreased by 14%–29%, and the number of insulin infusions also dropped. Furthermore, a Euclidean distance of 2–6 mg/dL·min confirmed a very high match between the simulated trajectory and the actual blood glucose levels. These improvements, while striking, must be interpreted cautiously given the small sample (n = 15), the short 14-day observation period, the absence of a control arm, and the lack of a closed-loop human trial; the reported TIR range also spans an unusually wide baseline that warrants further methodological clarification in future studies. This study is the first to demonstrate that digital twin technology can feasibly manage blood glucose in elderly patients with T2DM who have multiple comorbidities and fragile physiological functions. The system extends automated insulin delivery (AID) technology to elderly patients with T2DM. At the same time, it provides a reference for constructing digital twins for other chronic diseases in the elderly, such as hypertension and heart failure. Small sample size, short follow-up duration, technical operational barriers for the elderly population, lack of closed-loop human trials, and the unresolved black-box nature of LSTM remain existing problems. In the future, large-scale, long-term clinical trials are needed to improve the model’s ability to handle variable factors in real-world settings, with the long-term goal of developing life-course digital twin systems (22).

A cross-case comparison of the three exemplars reveals a clear maturity gradient. The UVA/Padova simulator represents the most mature paradigm — it has achieved regulatory recognition and reproducible in silico results, yet remains constrained by rigid physiological assumptions and has never been deployed as a therapeutic device. The exDSS system illustrates an intermediate stage — technically innovative but validated only in silico, with an unclear path to real-world deployment. The HDT for elderly T2DM represents the frontier — closest to bedside application but limited by small sample size (n = 15), short follow-up (14 days), and unresolved black-box concerns of its LSTM core. This progression from “regulated tool” → “in silico prototype” → “small-cohort pilot” rather than the reverse highlights that the field’s principal bottleneck is no longer modeling capability but validation infrastructure. For ease of intuitive comparison, the application scenarios, technical architecture, core innovations, main outcomes, and validation strategies of the three cases are summarized in **Table 3**, with a head-to-head comparison across additional dimensions in the new **Table 1**.

**Table 3:** Representative DT-AI application cases in diabetes research.

Case	Application scenario	Technical architecture	Core innovation	Main outcomes	Validation strategy	References
UVA/Padova T1DM simulator	Artificial pancreas/AID algorithm testing	Multi-compartment ODE simulator with virtual cohorts and sensor-error modeling	FDA-recognized in silico platform for closed-loop control testing	Extended from single-meal to full-day simulation; supports preclinical algorithm evaluation	V1: in silico validation; regulator-recognized tool for a specified context of use	(18–20)
exDSS	Exercise decision support in T1DM	OHSU virtual patient population; physiological simulation; rule-based recommendations	Exercise-specific DT decision support	Improved simulated time-in-range and reduced hypoglycemia during standardized exercise scenarios	V1: in silico validation only	(21, 72–74)
Human digital twin for elderly T2DM	Personalized management of elderly patients with T2DM	IoMT + LSTM + Matrix Profile + LIME/XGBoost + LB-MPC	Integrates multimodal real-world data for elderly multimorbidity management	Reported TIR improvement and insulin-dose reduction, but in a small short-duration cohort	V3: small-cohort real-world observation; no closed-loop RCT	(22, 75)

## 5 Current challenges and limitations

### 5.1 Key technical bottlenecks

Complex models, such as deep neural networks and ensemble learning, despite their high accuracy, possess a critical drawback: their “black-box” nature results in poor interpretability. Since the models provide no explanation, neither physicians nor patients can grasp the decision-making logic. This issue significantly erodes confidence and remains a primary obstacle to genuine clinical implementation (11, 76).

### 5.2 Implementation barriers at the clinical translation level

#### 5.2.1 Regulatory pathways

At present, regulatory authorities recognize DT and *in silico* models principally as (i) complementary tools that may partially substitute for animal studies, and (ii) instruments supporting trial design, dose optimization, and risk assessment. The UVA/Padova simulator, endorsed by the FDA in 2008 for artificial pancreas testing, remains the paradigmatic example. Three regulatory concepts must be clearly distinguished: (i) regulatory qualification of DT models as tools of known reliability; (ii) use of virtual cohorts to complement human trial data; and (iii) the more exploratory concept of virtual control arms replacing human placebo groups. Current regulator expectations for DT validation evidence typically include analytical verification, biological plausibility, quantitative performance metrics, and context-of-use documentation, as outlined in the FDA guidance on Good Machine Learning Practice (GMLP) (77), the EMA Reflection Paper on the use of AI in the medicinal product lifecycle (78), and the ASME V&V 40 framework (79). We emphasize that most current DT applications in diabetes remain at the simulation-validation stage, and only the UVA/Padova system has crossed into regulator-recognized use — a distinction we have carefully preserved throughout the manuscript to avoid overstating clinical readiness.

#### 5.2.2 Ethical, legal, and governance considerations

DT-AI models present many ethical and moral challenges (80). Digital twins require the collection and processing of highly sensitive health information, leading to a sharp increase in the risk of patient privacy breaches and data security issues. This heightens patient concerns and poses obstacles to clinical adoption (11, 81). Patients must retain meaningful autonomy over their health decisions. However, when algorithmic predictions are erroneously perceived as definitive, patients’ decision-making agency is insidiously eroded, devolving into a novel form of digital medical paternalism (82, 83). Furthermore, the use of DT-AI is supposed to provide everyone with equitable opportunities for health. In reality, however, it may do the opposite: it can widen existing health disparities, causing vulnerable groups to face greater obstacles in accessing medical care, disease prevention, and treatment outcomes (82). Ultimately, in complex and uncertain clinical situations, human ethical judgment, empathy, and reflective clinical reasoning cannot be fully replaced by artificial intelligence. These concerns must be considered against the backdrop of concrete regulatory and normative frameworks: the WHO Ethics and Governance of AI for Health guidance (84), the EU AI Act (2024), which classifies medical AI as high-risk (85), and the FDA’s Predetermined Change Control Plan guidance for adaptive algorithms (86). Professional bodies, including the American Medical Informatics Association and endocrinology societies, have also begun to issue position statements on responsible clinical AI adoption, which reinforce the need for transparent data-governance policies specific to diabetes DT-AI systems.

#### 5.2.3 Implementation and equity challenges

Real-world implementation faces additional barriers that are often underappreciated in technical reviews: (i) interoperability across electronic health record (EHR) systems, which remains fragmented across institutions and vendors; (ii) reimbursement models that do not yet recognize DT-based decision support as a billable clinical activity, creating a structural disincentive for adoption; (iii) digital literacy gaps among elderly and low-resource populations — particularly acute for diabetes, whose prevalence is highest in exactly these groups; (iv) computational infrastructure requirements that may exclude low- and middle-income settings, where the diabetes burden is growing fastest; and (v) workforce readiness, as most clinicians have not received systematic training in interpreting DT-AI outputs. Without proactive attention to equity, DT-AI risks widening rather than narrowing global diabetes disparities, undermining the very promise of “precision for all” that motivates the field.

### 5.3 Future perspectives

Building on the gaps identified above, we propose a tiered roadmap organized by translational horizon. Short-term (1–3 years): (i) standardize reporting requirements for DT-AI diabetes studies analogous to TRIPOD-AI and CONSORT-AI so that dataset, validation strategy, and reported limitations are transparently disclosed; (ii) prioritize prospective, closed-loop human validation of pilot systems such as HDT and exDSS; (iii) actively publish negative results and failure modes to counter publication bias. Mid-term (3–5 years): (iv) advance hybrid mechanistic–data-driven models — including physics-informed neural networks — to reduce data hunger while preserving physiological fidelity (38); (v) build federated-learning consortia across endocrinology centers to enable privacy-preserving multi-site training (87); (vi) develop XAI benchmarks tailored to clinical endocrinology so that explainability claims can be evaluated objectively. Long-term (>5 years): (vii) refine regulatory pathways for virtual control arms and continuous-learning DT systems (88); (viii) clarify medico-legal liability frameworks for AI-assisted clinical decisions; (ix) embed ethical deliberation, human-led decision frameworks, and equity audits at the design stage to prevent widening of health disparities (34, 82, 89, 90). Underpinning all three horizons is the principle that DT-AI should act as a clinical partner rather than a replacement, delivering fairer, more accessible, and more efficient diabetes care.

## Glossary

ADA: American Diabetes Association
ADME: Absorption, Distribution, Metabolism, Excretion
AID: Automated Insulin Delivery
AI: Artificial Intelligence
AMIA: American Medical Informatics Association
ASME: American Society of Mechanical Engineers
CGM: Continuous Glucose Monitoring
CKD: Chronic Kidney Disease
CONSORT-AI: Consolidated Standards of Reporting Trials–Artificial Intelligence
DPP-4: Dipeptidyl Peptidase-4
DR: Diabetic Retinopathy
DT: Digital Twin
DT-AI: Digital Twin–Artificial Intelligence (cross-fusion)
her: Electronic Health Record
EMA: European Medicines Agency
EU: European Union
exDSS: Exercise Decision Support System
FDA: U.S. Food and Drug Administration
FPG: Fasting Plasma Glucose
GDM: Gestational Diabetes Mellitus
GMLP: Good Machine Learning Practice
GLP-1R: Glucagon-Like Peptide-1 Receptor
GPCR: G Protein-Coupled Receptor
HbA1c: Glycated Hemoglobin
HDT: Human Digital Twin
IDF: International Diabetes Federation
IoMT: Internet of Medical Things
LB-MPC: Learning-Based Model Predictive Controller
LIME: Local Interpretable Model-agnostic Explanations
LLM: Large Language Model
LSDT: Large Language Model–Augmented Semantic Digital Twin
LSTM: Long Short-Term Memory
MHRA: Medicines and Healthcare products Regulatory Agency
ODE: Ordinary Differential Equation
OGTT: Oral Glucose Tolerance Test
PBPK: Physiologically Based Pharmacokinetics
PDE: Partial Differential Equation
PINN: Physics-Informed Neural Network
PK/PD: Pharmacokinetics/Pharmacodynamics
PRISMA-ScR: Preferred Reporting Items for Systematic Reviews and Meta-Analyses extension for Scoping Reviews
QSP: Quantitative Systems Pharmacology
RCT: Randomized Controlled Trial
SGLT2: Sodium-Glucose Cotransporter-2
SU: Sulfonylurea
T1DM: Type 1 Diabetes Mellitus
T2DM: Type 2 Diabetes Mellitus
TIR: Time in Range
TRIPOD-AI: Transparent Reporting of a Multivariable Prediction Model for Individual Prognosis or Diagnosis–Artificial Intelligence
TZD: Thiazolidinedione
UVA/Padova: University of Virginia/Padova
WHO: World Health Organization
XAI: Explainable Artificial Intelligence.
